# Transjugular intrahepatic portosystemic shunt with ePTFE-covered stentgrafts: incidence and predictors of shunt dysfunction

**DOI:** 10.1186/s13244-025-02122-2

**Published:** 2025-11-05

**Authors:** Michael B. Pitton, Lukas Müller, Fabian Stoehr, Arndt Weinmann, Christian Labenz, Jens Mittler, Roman Kloeckner, Christoph Düber, Gerd Otto

**Affiliations:** 1 Department of Díagnostic and Interventional Radiology, University Medical Center Mainz, Mainz, Germany; 2 Department of I. Medicine, University Medical Center Mainz, Mainz, Germany; 3 Department of General, Visceral and Transplant Surgery, University Medical Center Mainz, Mainz, Germany; 4 Institute of Interventional Radiology, University Hospital Schleswig-Holstein, Luebeck, Germany; 5 Hepatobiliäry and Transplant Surgery, University Medical Center Mainz, Mainz, Germany

**Keywords:** Transjugular intrahepatic porto-systemic shunt, Stent, Shunt dysfunction, Revision

## Abstract

**Objectives:**

To analyze revision rates after transjugular intrahepatic portosystemic shunt (TIPS) using expanded polytetrafluoroethylene-covered stentgrafts and to identify predictors of shunt revisions.

**Materials and methods:**

This single-center retrospective study included 514 consecutive patients (mean age 56.9 ± 12.7 years; 194 females) with TIPS placement between 2003 and 2021. Follow-up included clinical assessment, laboratory testing, ultrasound, and computed tomography. Reinterventions were categorized by type and technique. Univariable and multivariable Cox regression analyses were performed to identify predictors of shunt dilation and reduction.

**Results:**

A total of 149 patients (28.9%) required TIPS revision: 95 (18.5%) shunt dilation, 42 (8.2%) shunt reduction, and 12 (2.3%) others. Median time to first revision was 2.8 months (3.2 months for dilation, 1.9 months for reduction). Indications for first shunt dilation were persistent or recurrent refractory ascites (*n* = 61), recurrent variceal bleeding (*n* = 7), and asymptomatic stenosis or occlusion of the TIPS tract (*n* = 27). Indications for shunt reduction were hepatic encephalopathy refractory to conservative measures (*n* = 39) and acute liver failure following TIPS (*n* = 3). Forty-seven patients (9.1%) underwent two or more reinterventions. Multivariable Cox analysis identified immediate post-TIPS portosystemic pressure gradients > 8 mmHg, prior hepatic encephalopathy, and hepatorenal syndrome prior to TIPS as predictors of mandatory shunt dilation. In contrast, age ≥ 65 years, female gender, serum sodium levels, and a pre-TIPS hepatic hydrothorax were predictive of shunt reduction during revision.

**Conclusion:**

Around one in three patients requires shunt revision. Predictive factors for revision varied by intervention type: shunt dilation was linked to disease severity and portal pressure, whereas reduction was more closely related to the patient’s age and gender.

**Critical relevance statement:**

Patients who undergo TIPS require structured, long-term follow-up to identify clinical situations that may necessitate shunt adaptation or other secondary interventions.

**Key Points:**

- Shunt revision after TIPS occurs in one-third of patients, with prognostic significance.
- Several independent prognostic factors for both shunt dilation and reduction were identified.
- Structured long-term follow-up is crucial to identify patients needing shunt revision.

**Graphical Abstract:**

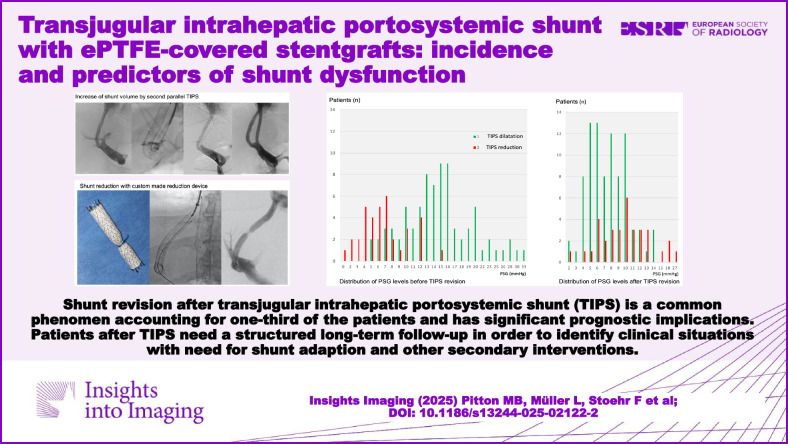

## Introduction

Over the past 20 years, the introduction of expanded polytetrafluoroethylene (ePTFE)-covered stents has significantly contributed to the widespread adoption of transjugular intrahepatic portosystemic shunt (TIPS), primarily due to a reduced incidence of stent dysfunction [1, 2]. However, there remains a risk of potential complications related to shunt function during follow-up, including both overfunction and underfunction of TIPS [3–6]. Previous studies have reported stenosis or occlusion of TIPS in over 10% of cases [7]. Additionally, the rate of revisions due to refractory hepatic encephalopathy (HE) has been reported to range between 5 and 13% of patients [7–10]. In such cases, TIPS revision is typically necessary, and several procedural options have been established [9–21]. Few studies have focused specifically on reinterventions and their associated risk factors [7–10]. As their focus was mainly on the first revision after TIPS, successive revisions during the long-term course are not reported in detail. The aim of this study was to investigate reintervention rates after TIPS using ePTFE-covered stent grafts during long-term follow-up. Beyond that, we aimed to identify risk factors related to mandatory TIPS revision and report on the respective interventional techniques.

## Materials and methods

### Patients

**Fig. 1:**
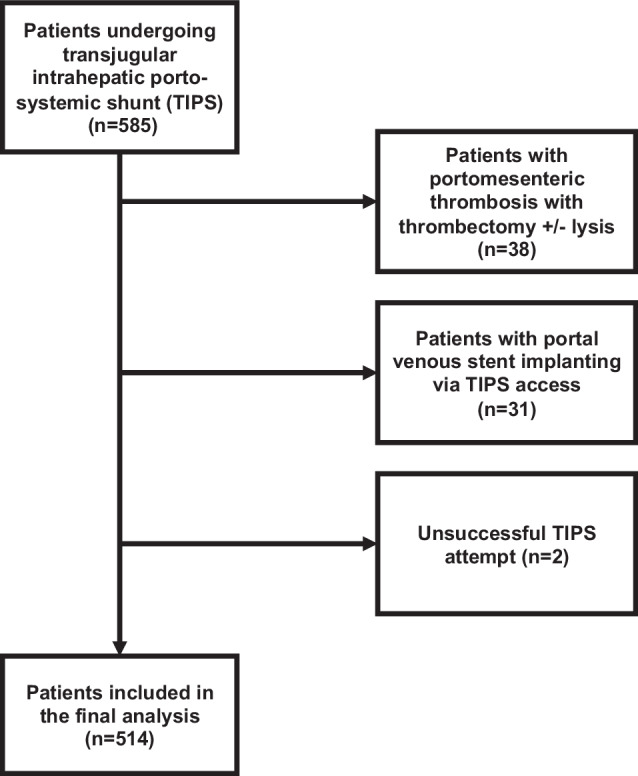
Flowchart of the patient selection process for this study

Between 2003 and 2021, a total of 585 TIPS were performed for porto-mesenterical pathologies (Fig. 1). Patients were eligible for inclusion if they were ≥ 18 years of age, had complete clinical and imaging data available, and underwent successful TIPS implantation primarily for portal hypertension-related indications. Exclusion criteria were unsuccessful TIPS attempt (*n* = 2), isolated portal venous stent placement via TIPS access without functional shunting (*n* = 31), and cases with primary thrombectomy and/or lysis due to portomesenteric thrombosis (*n* = 38). Patients with complete thrombosis of the portal vein, recanalization and stent treatment were excluded from further analysis. In two cases (0.3%), no TIPS implementation could be performed. Patients with early post-TIPS death were included. This resulted in a total of 514 patients (320 male, 194 female, age 56.9 ± 12.7 years (range 18–86)) for further analysis (Fig. 1). Patients’ baseline characteristics are provided in Table 1. For more details, we provide a separate table for cirrhotic and Budd-Chiari cases (Supplementary Table 1). Follow-up included all clinical reports and reinterventions from our institutional databases, all records from referring physicians or other hospitals, and registration offices. The study was carried out according to the Helsinki Declaration. The protocol was approved by the local ethics committee (No. 2022-16427_2, Rhineland-Palatinate, Germany), which waived the need for informed consent.

**Table 1:** Patient characteristics and clinical indication for TIPS

Baseline characteristics	All patients (*n* = 514)	1. Revision: dilatation (*n* = 95)	1. Revision reduction (*n* = 42)
Female, *n* (%)	194 (37.6)	35 (36.8)	25 (59.5)
Etiology of liver disease, *n* (%)			
Alcoholic	303 (58.9)	45 (30.2)	18 (12.1)
Viral hepatitis	45 (8.8)	6 (4.0)	6 (4.0)
Budd-Chiari syndrome	34 (6.6)	11 (7.4)	3 (2.0)
Biliary cirrhosis	13 (2.5)	1 (0.7)	0
Combinations of these	46 (8.9)	8 (5.4)	5 (3.4)
Cryptogenic etiology	42 (8.2)	6 (4.0)	5 (3.4)
Others	30 (5.8)	14 (9.4)	4 (2.7)
Clinical presentation and findings, *n* (%)			
CHILD A/B/C	36 (7.0)/340 (66.1)/138 (26.8)	6 (4.0)/58 (38.9)/5 (3.4)	3 (2.0)/33 (22.1)/3 (2.0)
MELD ≤ 14/ > 14*	327 (63.6)/187 (36.4)	20 (13.4)/29 (19.5)	8 (5.4)/16 (10.7)
History of hepatorenal syndrome	158 (30.6)	33 (22.1)	12 (8.1)
History of spontaneous bacterial peritonitis	98 (19.0)	21 (14.1)	7 (4.7)
History of hepatic encephalopathy	100 (19.4)	24 (16.1)	10 (6.7)
Budd-Chiari syndrome	52 (10.1)	8 (5.4)	1 (0.7)
Hepatocellular carcinoma	31 (6.0)	4 (2.7)	2 (1.3)
Cholangiocarcinoma	2 (0.4)		
Other tumors	23 (2.3)	1 (0.7)	2 (1.3)
Portal vein thrombosis	59 (11.4)	14 (9.4)	4 (2.7)
Hypersplenic syndrome (platelet count < 100.000/µL)	167 (32.4)	29 (19.5)	16 (10.7)
History of liver transplantation before TIPS	11 (2.1)		1 (0.7)
Liver transplantation during follow-up after TIPS	58 (11.2)	16 (10.7)	7 (4.7)
Renal failure	195 (37.8)	35 (23.5)	21 (14.1)
Clinical indication for TIPS, *n* (%)			
Variceal bleeding**	138 (26.8)	22 (14.8)	9 (6.0)
Refractory ascites***	371 (72.2)	70 (47.0)	32 (21.5)
Liver failure in Budd-Chiari	5 (1.0)	2 (1.3)	0

In addition to these patients, there was a smaller subgroup of revision cases (*n* = 12/149) that could not be clearly assigned to either of the two main groups (e.g., no measure and only TIPS control or embolization of esophageal and/or gastric varices). However, due to the small sample size, this subgroup is not reported separately

*TIPS* transjugular intrahepatic portosystemic shunt, *MELD* Model for End-stage Liver Disease

* According to the current EASL clinical practice guidelines on: liver transplantation a MELD Score > 14 is an indication for transplantation listing, as expected survival is less than 1 year without transplantation

** *n* = 19 of those patients had additionally refractory ascites

*** *n* = 74 of those patients had also had variceal bleeding in their history

### TIPS technique

The TIPS procedure was performed via transjugular access using a 10 F sheath advanced into the hepatic vein, followed by portal vein puncture through the liver parenchyma (RUPS-100, Cook) [22]. In selected cases, alternative access routes (e.g., from the left or middle hepatic vein or directly from the vena cava in Budd-Chiari syndrome) were used. The tract was dilated and stented using ePTFE-covered stent grafts (Viatorr®, Gore) extending from the portal end through the hepatic vein to the inferior vena cava. Since the stent graft is only available in a maximal length of 8 cm, two stent grafts were implanted in a telescopic technique in cases of longer TIPS tracts in order to achieve the above-mentioned extension. Portal and systemic venous pressures were measured simultaneously using a pigtail catheter in the main portal vein and the sheath in the right atrium to calculate the PSG. In patients with acute variceal bleeding or a history of variceal bleeding, respective varices were embolized during the TIPS procedure at the operator’s discretion. All procedures followed a standardized protocol by a single experienced operator. Post-intervention, patients received anticoagulation for at least 3 months; lifelong therapy was maintained in patients with Budd-Chiari syndrome or relevant coagulopathies.

TIPS revisions were indicated according to the clinical symptomatology and follow-up findings with ultrasound and CT. At our institution, patients are followed up at 3-month intervals after discharge. During each follow-up visit in outpatient care, a clinical examination, laboratory testing, and an ultrasound assessment of the TIPS are performed. Contrast-enhanced CT was used for confirmation of any pathological finding since Color Doppler ultrasound has shown limited accuracy [23]. In the current study, therefore, all indications for shunt revision were based on CT morphology and shunt angiography, including portal pressure measurements. In cases with insufficient release or new onset of hydropic decompensation, recurrent variceal bleeding, impaired liver function, hepatic encephalopathy or combinations of these, a TIPS angiography and pressure gradients of the TIPS tract were obtained. Based on the clinical, pathophysiologic, and angiographic findings, shunt dilation or shunt reduction was indicated, including additional stentgrafts for TIPS extension, stents for alignment of the TIPS tract, and diverse techniques for shunt reduction, respectively. Indications for first shunt dilation were recurrence of ascites, insufficient resolution, or persistence of ascites with PSG > 8 mmHg but without structural findings of the TIPS tract, circumscribed TIPS stenoses or occlusions, and recurrent bleeding events after TIPS. Clinical indication for shunt reduction was HE, refractory to medical treatment or acute liver failure after TIPS.

### Statistical methods

Statistical analysis was performed using SPSS, version 24 (IBM). Analysis included descriptive demographic data, clinical status, e.g., comorbidities, laboratory findings, and outcome data. Cox analysis was used to identify risk factors from pre-TIPS baseline data for respective TIPS revisions. In this analysis, various parameters in the categories patient demographics, liver disease severity and function, decompensation and complications, coagulation and hematological parameters, laboratory parameters as well as comorbidities were included. Survival data were analyzed using Kaplan–Meier curves, and strata were compared with log-rank testing. Patients who underwent liver transplantation were censored at the time of transplantation. Significant risk factors from the univariate analysis were included in a multivariate Cox analysis and were calculated using the LogRank tests. *p*-values ≤ 0.05 were rated significant. Given the exploratory nature of the study, no adjustment for multiple testing was done.

## Results

Three-day and 30-day mortality rates were 0.7% (*n* = 3) and 6.4% (*n* = 29) in elective cases and were 15.9% (*n* = 10) and 31.7% (*n* = 20) in emergencies, respectively. The 3-day mortality in elective cases was caused by peri-interventional intra-abdominal bleeding with liver failure in two cases, and one patient suffering from septicemia and renal failure in a preexisting spontaneous bacterial peritonitis (SBP). The complications of the procedure were graded according to the CIRSE recommendations as follows: Grade 1: *n* = 10 (1.9%), Grade 2: *n* = 4 (0.8%), Grade 3: *n* = 4 (0.8%), and Grade 6: *n* = 5 (1.0%). At the end of follow-up, 277 patients (53.9%) were alive. The median follow-up for the whole cohort was 13.6 months (IQR 2.9–42.2 months). For patients who were alive at the end of their follow-up, the median follow-up time was 20.0 months (IQR 4.0–52.9 months). For patients who died during follow-up, the median OS was 9.1 months (IQR 1.5–28.4 months). Overall survival rate at 1, 3, 5, and 10 years was 71%, 54%, 43.3%, and 29.4% respectively. Revision free survival at 1, 3, 5, and 10 years was 49.1%, 29.9%, 21.6%, and 13.3% respectively (Supplementary Fig. 1). Patients with TIPS dilation at the first revision presented an improved survival compared to those without (*p* < 0.001) (Supplementary Fig. 2). Patients with pre-TIPS history of HE presented an reduced overall survival compared to those without HE history before TIPS (27.9 versus 53.0 months, *p* = 0.011) (Supplementary Fig. 3a) as indicator of an overall reduced liver function. However, patients with post-TIPS refractory HE and respective revision had no difference in survival compared to those without (*p* = 0.420) (Supplementary Fig. 3b).

During follow-up, 149 of 514 patients (28.9%) received at least one TIPS revision, 47 of whom (9.1%) with two or more reinterventions, resulting in 243 revision procedures (Table 2) covering a total of 380 particular interventional measures (Table 3). There were 15 patients with opposing measures during follow-up, meaning that 8 of 95 cases (8.4%) with TIPS dilation at first revision received TIPS reduction at a successive revision during follow-up. Vice versa, 7 of 42 cases with TIPS reduction at first revision received a dilatation of the TIPS tract at a successive revision. Median interval to the first revision was 2.8 months (IQR 1.1–14.8 months), 3.2 months (IQR 1.0–18.00 months) for first shunt dilation, and was 1.9 months (IQR 1.0–4.3 months) for first shunt reduction.

**Table 2:** Number and types of TIPS revisions

Number of TIPS revisions	1.	2.	3.	4.	5.	6.	7.
Patients, *n* (%)	149 (28.9)	47 (9.1)	21 (4.1)	12 (2.3)	8 (1.6)	4 (0.8)	2 (0.4)
TIPS dilatation, *n* (%)	95 (18.5)	28 (5.4)	15 (2.9)	10 (1.8)	4 (0.8)	3 (0.6)	1 (0.2)
TIPS reduction, *n* (%)	42 (8.2)	12 (2.3)	4 (0.8)		1 (0.2)		
TIPS control	8 (1.6)	6 (1.2)	2 (0.4)	2 (0.4)	2 (0.4)		
Other revision, *n* (%)	4 (0.8)	1 (0.2)			1 (0.2)	1 (0.2)	1 (0.2)
Time interval after TIPS							
Median (months)	2.8	10.4	21.9	21.6	22.0	16.7	79.1
Min–max (months)	0–140	0.2–161	0.4–124	3.5–124	6.7–155	8.0–130	11.7–146
Time between revisions							
Median (months)		4.0	5.2	2.9	0.7	2.9	9.5
Min–Max (months)		0.2–114	0.2–43.9	0–34.3	0.1–51.4	0–16	2.6–16.5

Time intervals until the 1st TIPS revision and between successive TIPS revisions

*TIPS* transjugular intrahepatic portosystemic shunt

**Table 3:** Intervention techniques, number and types of devices used for shunt adaptation

Devices for shunt dilation	*n*
TIPS dilation with POBA only	57
TIPS dilation with cutting balloon	2
TIPS extension with Viatorr® stentgrafts	60
Self-expandable stents, Absolute®, Luminexx®, Smart control®, Sinus XL®	39
Balloon expandable stents, Dynamic®, Palmaz P308®,	39
Thrombectomy, mazeration, aspiration, Rotarex®	36
Thrombolysis local, Actilyse®	2
Parallel TIPS implantation	3
Devices for shunt reduction	
Sinus Reduction Stent®, dedicated reduction device	45
Viatorr®, Fluency®, Viabahn® Wallgraft® stentgrafts	23
Embolization of stent interspatium	10
Amplatzer® device for complete occlusion	15
Custom-made reduction device	6
Extraction of a Viatorr® and replacement of a Viatorr®	1
No mechanical measures to the TIPS Tract	20
Embolization of esophageal and/or gastric varices	14
Other measures	8
Total measures	380

Company information: Viatorr®, VIATORR® Controlled Expansion and Viabahn®, Gore and Associates, Flagstaff, AZ, USA; Absolute®, Abbott, North Chicago, IL, USA; Luminexx® and Fluency®, Bard, Murray Hill, NJ, USA; Smart control® and Palmaz P308®, Cordis, Santa Clara, CA, USA; Sinus XL®, Optimed, Ettlingen, Germany; Dynamic®, Biotronik, Berlin, Germany; Rotarex®, Straub Medical, Wangs, Switzerland; Actilyse®, Boehringer Ingelheim, Ingelheim, Germany; Wallgraft®, Boston Scientific, Marlborough, MA, USA

*POBA* plain old balloon angioplasty, *TIPS* transjugular intrahepatic portosystemic shunt

**Fig. 2:**
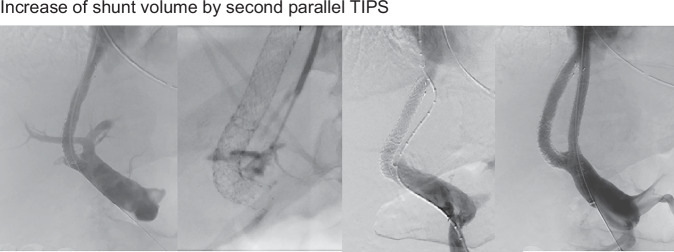
Creation of a second TIPS tract parallel to the first one in order to increase cross cross-sectional area of the TIPS for an increase in the shunt volume

Clinical indications for first shunt dilation (group I) were recurrence of ascites, insufficient resolution, or persistence of ascites with PSG > 8 mmHg but without structural findings of the TIPS tract (*n* = 46), circumscribed TIPS stenoses or occlusions (*n* = 15), and recurrent bleeding events after TIPS (*n* = 7). The rate of clinical TIPS dysfunction with overt clinical symptoms was 68 of 514 patients (13.2%). There were another 5.3% of cases (27 of 514) with circumscribed shunt stenosis (*n* = 13) or complete occlusions (*n* = 14) who remained clinically asymptomatic and received a service intervention to maintain shunt function. Shunt dilation or recanalisation covered diverse techniques and combinations of those, which are depicted in Table 3. One of these techniques was the implantation of a second TIPS, which was mandatory in 3 cases (Table 3, Fig. 2). Immediately before shunt dilation, the median PSG was 14.5 mmHg (range 5–33 mmHg) and was significantly reduced to a median PSG of 7.0 mmHg (range 2–14 mmHg, *p* < 0.001) after shunt dilation. The respective patients (*n* = 95) presented an only slightly higher initial post-TIPS PSG compared to those without need for secondary shunt dilation (7 mmHg median (range 0–15 mmHg) versus 6 mmHg median (range 0–17 mmHg, *p* < 0.001)). Univariable Cox analysis identified nine potential baseline risk factors for secondary shunt dilation. The multivariable analysis yielded history of hepatorenal syndrome (HRS) and HE and post-TIPS PSG > 8 mmHg as significant predictors for first shunt dilation (Table 4, detailed overview of the distribution of the factors in Supplementary Table 2).

**Table 4:** Univariate and multivariate Cox analysis on risk factors for first revision with shunt dilation

	Univariate Cox analysis				Multivariate Cox analysis*			
Risk factors for first revision with shunt dilation	*p*-value	HR	95.0% CI		*p*-value	HR	95.0% CI	
Age at time of TIPS	0.036	0.983	0.967	0.999				
Female	0.249	0.781	0.513	1.189				
Hydropic decompensation	0.170	1.618	0.814	3.217				
Refractory ascites	0.123	1.718	0.864	3.415				
Refractory hydrothorax	0.627	1.170	0.622	2.198				
Variceal bleeding	0.355	0.821	0.541	1.247				
Emergency TIPS	0.476	1.257	0.670	2.359				
Budd-Chiari syndrome	0.397	1.265	0.735	2.177				
CHILD score	0.017	1.171	1.029	1.333				
MELD score	0.071	1.038	0.997	1.081				
NaMELD score	0.012	1.050	1.011	1.090				
Hepatorenal syndrome	0.036	1.577	1.031	2.413	0.050	1.533	0.999	2.352
Spontaneous bacterial peritonitis	0.173	1.404	0.862	2.287				
Hepatic encephalopathy	0.011	1.833	1.147	2.930	0.017	1.775	1.110	2.838
Portal vein thrombosis (non-occlusive)	0.282	1.366	0.774	2.411				
Pressure gradient (PSG) pre-TIPS (mmHg)	0.016	1.043	1.008	1.078				
PSG post-TIPS (mmHg)	< 0.001	1.132	1.058	1.211				
∆PSG pre- post-TIPS (mmHg)	0.507	0.987	0.949	1.026				
PSG ratio post-TIPS/pre-TIPS < 0.5	0.063	0.583	0.330	1.031				
PSG post-TIPS > 8 mmHg	< 0.001	2.696	1.706	4.260	< 0.001	2.703	1.711	4.270
INR	0.037	2.324	1.051	5.136				
Creatinin (mg/dL)	0.405	1.110	0.868	1.419				
Bilirubin (mg/dL)	0.232	1.035	0.978	1.095				
Albumin (g/L)	0.166	0.977	0.946	1.010				
Sodium (mmol/L)	0.158	0.975	0.942	1.010				
Platelet count/nL	0.497	1.001	0.999	1.002				
Hypersplenic syndrome (platelet count < 100/nL)	0.691	1.093	0.705	1.695				
Renal function impaired	0.970	1.008	0.666	1.527				
Cardiac diseases	0.781	0.999	0.988	1.009				
Pulmonary diseases	0.628	1.132	0.684	1.874				
Arterial hypertension	0.460	0.838	0.523	1.341				
Diabetes mellitus	0.601	0.880	0.544	1.422				
Coagulopathy	0.226	1.439	0.799	2.594				
Hepatocellular carcinoma	0.795	0.876	0.321	2.389				
Other tumors	0.656	0.909	0.598	1.383				
Chronic pancreatitis	0.092	2.373	0.869	6.474				
Polyneuropathy	0.854	1.203	0.168	8.640				
Hypo-/hyperthyrodism	0.385	0.792	0.468	1.340				
Vasculitis	0.485	0.049	0.000	234.305				
Colonic polyps	0.306	0.357	0.050	2.561				

*TIPS* transjugular intrahepatic portosystemic shunt, *MELD* Model for End-stage Liver Disease, *PSG* pressure gradient

* Only statistically significant variables are reported here

Clinical indication for shunt reduction (group II) was HE, refractory to medical treatment. To this end, a combination of devices and measures was used (Table 3, Supplementary Figs. 4–6). Before shunt revision, the overall median PSG level was 6 mmHg (range 0–15 mmHg) and was effectively increased to a median PSG of 10 mmHg (range 2–27 mmHg) after shunt reduction, *p* < 0.001. Univariable Cox analysis identified six potential risk factors. Interestingly, the aggregated scores and markers for impaired liver function (e.g., Model for End-Stage Liver Disease (MELD) score, Child score, pre-TIPS- HRE, -SBP, or -HE), emergency cases, variceal bleeding, or refractory ascites were no significant predictors for post-TIPS shunt reduction because of refractory HE. The multivariable Cox analysis confirmed age, female gender, pre-TIPS Serum-Sodium, and hydrothorax before TIPS as independent predictors of refractory shunt reduction for HE (Table 5, detailed overview of the distribution of the factors in Supplementary Table 2).

**Table 5:** Univariate and multivariate Cox analysis on risk factors for first revision with need for shunt reduction

	Univariate Cox analysis				Multivariate Cox analysis*			
Risk factors for first revision with shunt reduction	*p*-value	HR	95.0% CI		*p*-value	HR	95.0% CI	
Age at time of TIPS	0.002	1.048	1.018	1.079	< 0.001	1.053	1.024	1.083
Female	0.008	2.294	1.238	4.250	0.003	2.595	1.392	4.836
Hydropic decompensation	0.885	0.938	0.395	2.227				
Refractory ascites	0.994	1.003	0.423	2.382				
Refractory hydrothorax	0.026	2.320	1.107	4.861	0.015	2.499	1.192	5.239
Variceal bleeding	0.664	0.871	0.467	1.624				
Emergency TIPS	0.172	0.043	0.000	3.939				
Budd-Chiari syndrome	0.266	0.513	0.158	1.665				
CHILD score	0.460	0.927	0.757	1.134				
MELD score	0.758	1.010	0.949	1.075				
NaMELD score	0.094	0.947	0.889	1.009				
Hepatorenal syndrome	0.924	1.033	0.528	2.021				
Spontaneous bacterial peritonitis	0.672	0.839	0.372	1.891				
Hepatic encephalopathy	0.318	1.437	0.706	2.925				
Portal vein thrombosis (non-occlusive)	0.690	0.811	0.289	2.272				
Pressure gradient (PSG) pre-TIPS (mmHg)	0.966	1.001	0.948	1.057				
PSG post-TIPS (mmHg)	0.108	0.912	0.816	1.020				
∆PSG pre- postTIPS (mmHg)	0.360	0.975	0.923	1.030				
PSG ratio post-TIPS/pre-TIPS < 0.5	0.133	24,207	0.381	1539.386				
PSG post-TIPS < 8 mmHg	0.393	1.567	0.559	4.394				
INR	0.206	0.373	0.081	1.723				
Creatinin (mg/dL)	0.115	1.301	0.938	1.803				
Bilirubin (mg/dL)	0.308	0.876	0.679	1.130				
Albumin (g/L)	0.602	1.013	0.965	1.064				
Sodium (mmol/L)	0.003	1.084	1.028	1.143	0.002	1.094	1.035	1.157
Platelet count/nL	0.087	0.997	0.993	1.000				
Hypersplenic syndrome	0.406	1.307	0.695	2.458				
Renal function impaired	0.166	1.534	0.837	2.811				
Cardiac diseases	0.842	0.998	0.984	1.014				
Pulmonary diseases	0.897	0.947	0.411	2.182				
Arterial hypertension	0.158	1.567	0.839	2.927				
Diabetes mellitus	0.019	2.084	1.128	3.852				
Hypertensive gastropathy	0.495	0.792	0.405	1.548				
Coagulopathy	0.982	0.988	0.352	2.773				
Hepatocellular carcinoma	0.856	0.877	0.212	3.633				
Other tumors	0.758	1.068	0.704	1.621				
Chronic pancreatitis	0.531	0.048	0.000	630.231				
Polyneuropathy	0.680	0.049	0.000	83,390.009				
Hypo-/hyperthyrodism	0.203	1.434	0.823	2.497				
Vasculitis	0.026	5.053	1.218	20.970				
Colonic polyps	0.447	0.048	0.000	122.894				

* Only statistically significant variables are reported here

*TIPS* transjugular intrahepatic portosystemic shunt, *MELD* Model for End-Stage Liver Disease, *PSG* pressure gradient

**Fig. 3:**
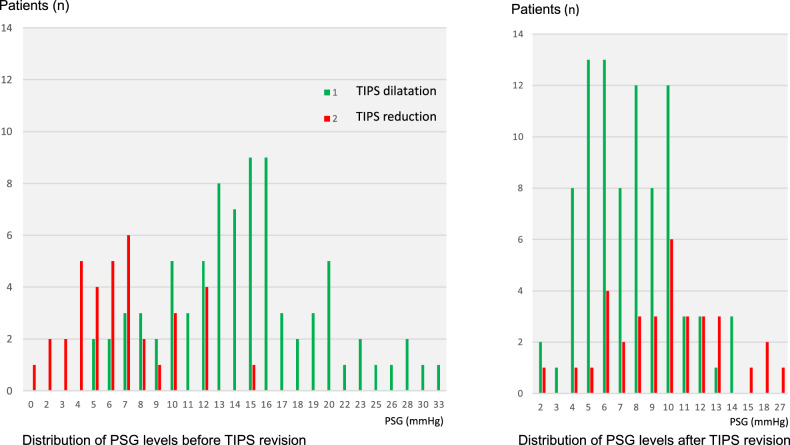
Distribution of PSG levels before and after TIPS revision

PSG levels before revision were significantly different in the patients with shunt dilation (range 5–33 mmHg) and shunt reduction (range 0–15 mmHg, Fig. 3, *p* < 0.001). However, there was a wide range and a remarkable overlap of pressure levels between both groups. Depending on the clinical presentation, e.g., a distinct PSG level of 10 mmHg could therefore indicate either further shunt dilatation in case of hydropic decompensation or, vice versa, for shunt reduction in refractory HE (Fig. 3). After revision, there was an approximation of PSG levels between both groups (group I: range 2–14 mmHg versus group II: range 2–27 mmHg).

## Discussion

The technique of TIPS creation has been refined over the past decades. In particular, ePTFE-covered stent grafts, now considered the standard of care, have improved patency rates and clinical outcomes in cases of variceal bleeding and refractory ascites [24–29]. Here, we present our single-center data on the use of ePTFE-covered stent grafts for TIPS and analyze the clinical conditions, as well as the frequency and types of TIPS revisions during long-term follow-up.

In this study, the majority of revisions occurred within the first year. Our data identified post-TIPS PSG > 8 mmHg, a history of HRS, and HE prior to TIPS creation as independent predictors of revisions requiring shunt dilation or recanalization. The indication for shunt dilation included both TIPS revisions in symptomatic patients (13.2%) as well as asymptomatic patients with TIPS stenosis or occlusions (5.3%). These cases were identified through structured follow-up visits and would not have been detected based on clinical symptoms alone, highlighting the importance of structured follow-up after TIPS [26]. In about half of the cases requiring shunt dilation, no circumscribed structural changes were detected. One explanation might be that the compliance of the surrounding liver tissue decreases with progressive liver cirrhosis, counteracting the radial force of the elastic stent grafts and causing some imperceptible diameter reduction with a gradual increase in PSG over time. Another explanation could be the angulation at the entry site of the Viatorr stent grafts into the portal vein, which may have resulted in overstretching of the convex side of the stent graft and, conversely, compression of stent struts and pleating of the membrane on the concave endoluminal surface. Such irregularities in stent struts and membrane may increase the pressure gradient, but they are difficult to detect by CT or shunt angiography.

There is a broad armamentarium for recanalization [30–32]. Moreover, thrombolysis might be indicated in TIPS thrombosis [33, 34] or Laser recanalization in chronically occluded TIPS tracts [35]. There is still a debate on whether anticoagulation prevents TIPS stenosis or occlusions. Previous studies concluded that continuous anticoagulation for 30 days post-TIPS therapy can effectively prevent stent stenosis or occlusion [36, 37].

The revision rate for refractory HE reported in this study (8.2%) is in line with reported data from the literature (5–13%) [7–10]. The median time to refractory HE was 1.9 months, occurring earlier compared to shunt dilations. Multivariable Cox analysis identified age, female gender, sodium level, and a history of hydrothorax as independent risk factors. Previous studies have reported older age, low serum sodium, low albumin, high creatinine, shunt size, and a history of HE prior to TIPS as independent predictors [8, 9]. Notably, in our cohort, a history of HE prior to TIPS did not significantly increase the rate of post-TIPS refractory HE. This is consistent with Saab et al, who concluded that controlled pre-TIPS HE did not negatively impact clinical outcomes after TIPS [11]. The extent of portosystemic shunt volume seems to play an important role in the pathophysiology of both spontaneous HE before TIPS, primary HE post-TIPS, and refractory post-TIPS HE [16, 17]. As such, our standard procedure includes occluding all obvious preexisting ectopic portosystemic shunts during the initial TIPS procedure, leaving the shunt volume primarily regulated by the diameter of the TIPS tract. Some authors have suggested underdilating stent grafts during TIPS creation to allow for a slower adaptation of portal hemodynamics and better preservation of liver function [18, 19]. Vice versa, this might, however, increase the reintervention rate with the need for more shunt dilation during revision. Numerous techniques for TIPS shunt reduction have been reported [9, 20, 21, 38]. In this study, we predominantly used dedicated reduction devices for cases with moderate urgency. These devices take some time to induce thrombotic obliteration between the initial TIPS stent graft and the reduction device, leading to a delayed pressure increase. For cases of high-grade HE and high urgency, we used either custom-made devices or a combination of stent grafts with coil deposits between both layers to create the desired shunt stenosis. Amplatzer devices were used for simple and effective shunt occlusion.

Setting the appropriate PSG remains a balancing act and must be tailored to each individual patient. For reliable hemodynamic assessment, a delayed PSG measurement 3–7 days after primary TIPS creation should be used [39]. Immediate post-TIPS PSG values reflect acute volume shifts occurring right after the TIPS shunt is opened and the subsequent adaptive reactions of the venous blood pool, right atrium, and pulmonary circulation. Delayed PSG values are considered more reliable because they reflect the equilibration to the new shunt hemodynamics [40, 41]. Our work demonstrates that various types of interventions are needed repeatedly and in varying numbers over the long term to maintain adequate TIPS function. In our opinion, a strict follow-up policy is crucial for adjusting shunt volume to ensure high patency rates and optimal TIPS function.

While all TIPS procedures in our cohort were performed using Viatorr® stent grafts with a standardized technique, we did not explicitly assess the impact of stent graft length on shunt dysfunction. However, clinical experience suggests that excessive extension into the portal or hepatic vein branches may influence flow patterns and predispose to dysfunction. Although our dataset did not allow for a systematic analysis of this parameter, future studies incorporating detailed documentation of stent dimensions and positioning could help clarify its role in shunt patency and clinical outcomes.

Regarding the prognostic outcome after TIPS, the survival rates reported for our cohort are comparable to previous mortality rates, which range between 20 and 40% after 1 year, particularly those reported recently for covered stent grafts by Büttner et al [42–45]. In their study, the authors reported overall TIPS dysfunction rates of 28.4%, 38.9%, and 52.2% at 1, 2, and 5 years of follow-up. The increased revision rates in their study might be attributed to the fact that the majority of their patients were treated with bare-metal stents (68.2%). The improved overall revision rate of 28.9% in our study can therefore be attributed to the exclusive use of ePTFE-covered stent grafts. This rate is comparable to that reported by Bureau et al, who demonstrated a 2-year patency rate of 76% for ePTFE stent grafts compared to 36% for uncovered prostheses [1].

Our study has several limitations. It is a retrospective analysis covering a long period of time. However, the data reflect a consistent approach to TIPS procedures with a uniform strategy. Clinical selection criteria and indications for TIPS may have evolved over time, such as the shift from conservative management of clinical decompensation before TIPS to the introduction of rifaximin for managing HE. Additionally, this is a single-center study. Variations in patient inclusion criteria, clinical stages, underlying causes of liver cirrhosis, comorbidities, and follow-up strategies between different centers could explain differences in the predictors for revision observed in other studies. Therefore, the predictors mentioned in our results, along with other clinical factors discussed, should be further investigated in prospective studies using more integrated risk models. Another limitation is the inclusion of patients with Budd-Chiari syndrome. This group represents a small subset and differs markedly from patients with cirrhosis due to their opposing coagulation profile. Nevertheless, these patients were included in our study to reflect clinical reality and because the same standardized TIPS procedure was applied. In the future, especially multicenter studies may provide further insights into revision rates in this specific patient population. In addition, our study included patients with small liver malignancies and those in the transplantation setting. The characteristics of these patients may differ from those of the broader cohort. In these cases, the decision to perform a TIPS was made by a dedicated transplantation board—comprising surgeons, internists, radiologists, and anesthesiologists—and was based on clinically significant portal hypertension as the leading indication. Another limitation of our study is that the analysis of risk factors for revision was restricted to the first revision only. This decision was based on the availability of a representative number of patients for this event. While our descriptive reporting of second and subsequent revisions may offer an initial insight, larger multicenter datasets will be essential to provide the next level of evidence. Future studies should therefore aim to include sufficient numbers of later revision events to enable robust risk factor analyses beyond the first revision. Another limitation is that our time-to-event analyses after TIPS revision (Supplementary Figs. 2, 3) might be subject to immortal time bias [46], a systematic error that occurs when a period during which the event cannot occur is incorrectly included in the analysis, which should be considered when interpreting the associated results. Furthermore, multivariable analyses were not performed for survival outcomes in the context of the revisions. As such, these results should be interpreted descriptively, and future studies with larger sample sizes are warranted to confirm their significance of these interesting aspects for clinical decision-making.

## Conclusion

In summary, our data identified post-TIPS PSG > 8 mmHg, a history of HRS, and HE prior to TIPS as independent predictors for TIPS dilation during follow-up. Predictors for refractory HE with mandatory TIPS reduction included female gender, older age, sodium level, and a history of refractory hydrothorax. Interestingly, a history of medically controlled HE prior to TIPS does not increase the rate of refractory HE after TIPS. Patients who undergo TIPS require structured long-term follow-up to identify clinical situations that may necessitate shunt adaptation and other secondary interventions.

## Supplementary information

ELECTRONIC SUPPLEMENTARY MATERIAL

## Data Availability

Data cannot be shared publicly because of institutional and national data policy restrictions imposed by the Ethics committee of the Medical Association of Rhineland Palatinate, Mainz and the data protection office of the Johannes Gutenberg-University Mainz, Germany since the data contain potentially identifying and sensitive patient information. Data are available upon reasonable request from the Johannes Gutenberg-University Mainz Medical Center (contact via radiologie-sekreteriat@unimedizin-mainz.de and datenschutz@unimedizin-mainz.de) for researchers who meet the criteria for access to confidential data.

## Abbreviations

CT: Computed tomography
ePTFE: Expanded polytetrafluoroethylene
HE: Hepatic encephalopathy
HRS: Hepatorenal syndrome
MELD: Model for End-stage Liver Disease
PSG: Porto-systemic pressure gradient
SBP: Spontaneous bacterial peritonitis
TIPS: Transjugular intrahepatic portosystemic shunt
