# Supplementary material for: Transjugular intrahepatic portosystemic shunt with ePTFE-covered stentgrafts: incidence and predictors of shunt dysfunction
Source: Insights Imaging. 2025 Nov 5;16:244. doi: 10.1186/s13244-025-02122-2 (PMC12589687; doi:10.1186/s13244-025-02122-2)
Supplement: Supplementary file 1 — ELECTRONIC SUPPLEMENTARY MATERIAL [file 13244_2025_2122_MOESM1_ESM.docx]

**Transjugular Intrahepatic Portosystemic Shunt with ePTFE-covered**

**stentgrafts: incidence and predictors of shunt dysfunction**

**ELECTRONIC SUPPLEMENTARY MATERIAL**

**Supplementary Table 1: Patient characteristics and clinical indication for TIPS in Non-BuddChiari and BuddChiari**

| Baseline characteristics | Non Budd Chiari  n=462 | % | Budd Chiari  n=52 | % |
| --- | --- | --- | --- | --- |
| Age (y) mean±SD (range) | 58.2±11.7 |  | 47.1±14.9 |  |
| Female | 165 | 35.7 | 29 | 55.8 |
| Etiology of liver disease |  | | | |
| Alcoholic | 293 | 63.4 | 10 | 19.2 |
| Viral hepatitis | 44 | 9.5 | 1 | 1.9 |
| Biliary cirrhosis | 11 | 2.4 | 2 | 3.8 |
| Combinations of these | 39 | 8.4 | 7 | 13.5 |
| Cryptogenic etiology | 39 | 8.4 | 3 | 5.8 |
| Others | 27 | 5.8 | 2 | 3.8 |
| Clinical presentation and findings |  | | | |
| CHILD A / B / C | 34/304/124 | 7.4/65.8/26.8 | 2/36/14 | 3.8/69.2/26.9 |
| MELD ≤14 / >14* | 290/172 | 62.8/37.2 | 37/15 | 71.2/28.8 |
| History of hepatorenal syndrome (HRS) | 150 | 32.5 | 8 | 15.3 |
| History of spontaneous bacterial peritonitis (SBP) | 89 | 19.3 | 9 | 17.3 |
| History of hepatic encephalopathy (HE) | 88 | 19.0 | 12 | 23.1 |
| Hepatocellular carcinoma (HCC) | 28 | 6.1 | 3 | 5.8 |
| Cholangiocarcinoma (CCC) | 2 | 0.4 | 0 | 0.0 |
| Other Tumors | 11 | 2.4 | 1 | 1.9 |
| Portal vein thrombosis | 50 | 10.8 | 9 | 17.3 |
| Hypersplenic syndrome (Platelet count <100.000/µl) | 159 | 34.4 | 8 | 15.3 |
| History of liver transplantation before TIPS | 10 | 2.2 | 1 | 1.9 |
| Liver transplantation during follow-up after TIPS | 53 | 11.5 | 5 | 9.6 |
| Renal failure | 185 | 40.0 | 10 | 19.2 |
| Clinical indication for TIPS |  | | | |
| Variceal bleeding** | 130 | 28.1 | 4 | 7.7 |
| Refractory ascites*** | 328 | 77.5 | 43 | 82.7 |
| Liver failure in Budd-Chiari | 0 | 0 | 5 | 9.6 |
| Varices | 4 | 8.7 | 0 | 0 |

*According to the current EASL clinical practice guidelines: liver transplantation a MELD-Score >14 is an indication for transplantation listing as expected survival is less than 1 year without transplantation. **n=19 of those patients had additionally refractory ascites. ***n = 74 of those patients had also had variceal bleeding in the history

**Supplementary Table 2: Riskfactors for Cox analysis**

| Distributiuon of risk factors n=514 | All (n=514) | % | 1^st^ TIPS Dilation (n=149) | % | 1^st^ TIPS Reduction (n=42) | % |
| --- | --- | --- | --- | --- | --- | --- |
|  |  |  |  |  |  |  |
| Age at time of TIPS | 56.9±12.7 |  | 55.9±12.2 |  | 61.8±10.3 |  |
| Female | 194 | 37.6 | 63 | 42.3 | 25 | 59.5 |
| Hydropic decompensation | 444 | 86.4 | 134 | 89.9 | 36 | 85.7 |
| Refractory ascites | 378 | 73.5 | 115 | 77.2 | 28 | 66.7 |
| Refractory hydrothorax | 65 | 12.6 | 22 | 14.8 | 9 | 21.4 |
| Variceal bleeding | 218 | 42.4 | 55 | 36.9 | 16 | 38.1 |
| Emergency TIPS | 63 | 12.3 | 12 | 8.1 | 0 | 0 |
| Budd Chiari syndrome | 52 | 10.1 | 21 | 14.1 | 3 | 7.1 |
| CHILD score | 8.8±1.6 |  | 8.8±1.6 |  | 8.5±1.6 |  |
| MELD score | 13.9±5.7 |  | 13.6±4.9 |  | 13.3±4.5 |  |
| NaMELD score | 17.5±5.9 |  | 17.1±5.2 |  | 15.6±4.6 |  |
| Hepatorenal syndrome (HRS) | 158 | 30.7 | 48 | 32.2 | 12 | 28.6 |
| Spontaneous bacterial peritonitis (SBP) | 98 | 19.1 | 30 | 20.1 | 7 | 16.7 |
| Hepatic encephaopathy (HE) | 100 | 19.5 | 37 | 24.8 | 10 | 23.8 |
| Portal vein thrombosis (non-occlusive) | 59 | 11.5 | 21 | 14.1 | 4 | 9.5 |
| Pressure gradient (PSG) pre TIPS (mmHg) | 17.6±5.5 |  | 18.5±5.5 |  | 17.8±5.2 |  |
| Pressure gradient (PSG) post TIPS (mmHg) | 5.7±2.8 |  | 6.2±2.8 |  | 5.2±2.7 |  |
| ∆PSG pre - postTIPS (mmHg) | -11.8±5.2 |  | -12.2±4.9 |  | -12.6±3.7 |  |
| PSG ratio post-TIPS/pre-TIPS <0.5 | 452 | 87.9 | 135 | 90.6 | 42 | 100 |
| PSG post TIPS >8 mmHg | 68 | 13.2 | 30 | 20.1 | 4 | 9.5 |
| INR | 1.3±0.2 |  | 1.3±0.2 |  | 1.2±0.2 |  |
| Creatinin (mg/dl) | 1.4±0.9 |  | 1.4±0.7 |  | 1.5±0.9 |  |
| Bilirubin (mg/dl) | 2.2±3.6 |  | 2.0±2.9 |  | 1.5±1.1 |  |
| Albumin (g/l) | 27.4±8.2 |  | 27.6±6.6 |  | 28.3±5.2 |  |
| Sodium (mmol/l) | 134.6±5.6 |  | 134.9±5.5 |  | 137.1±3.9 |  |
| Platelet count /nl | 158.0±110.5 |  | 162.9±124 |  | 137.1±84.3 |  |
| Hypersplenic syndrome (Platelet count <100/nl) | 167 | 32.5 | 50 | 33.6 | 15 | 35.7 |
| Renal function impaired | 195 | 37.9 | 62 | 41.6 | 20 | 47.6 |
| Cardiac diseases | 98 | 19.1 | 28 | 18.8 | 12 | 28.6 |
| Pulmonary diseases | 45 | 8.8 | 14 | 9.4 | 4 | 9.5 |
| Arterial hypertension | 154 | 30.0 | 46 | 30.9 | 16 | 38.1 |
| Diabetes mellitus | 150 | 29.2 | 47 | 31.5 | 18 | 42.9 |
| Coagulopathy | 40 | 7.8 | 18 | 12.1 | 4 | 9.5 |
| Hepatocellular carcinoma | 31 | 6.0 | 8 | 5.4 | 2 | 4.8 |
| Other tumors | 14 | 2.7 | 3 | 2.0 | 1 | 2.4 |
| Chronic pancreatitis | 12 | 2.3 | 4 | 2.7 | 0 | 0 |
| Polyneuropathy | 5 | 1.0 | 2 | 1.3 | 0 | 0 |
| Hypo- / Hyperthyrodism | 62 | 12.1 | 19 | 12.8 | 9 | 21.4 |
| Vasculitis | 5 | 1.0 | 2 | 1.3 | 2 | 4.8 |
| Colonic Polyps | 14 | 2.7 | 2 | 1.3 | 0 | 0 |

**Supp Figure 1.** Overall survival (A) and Revision-free survival (B).

**Supp Figure 2.** Overall survival of patients with and without shunt dilation at first revision

**Supp Figure 3:** (A) Overall survival in patients with preTIPS history of HE presented an reduced overall survival compared to those without HE history before TIPS (B) Overall survival in patients with postTIPS refractory HE and respective revision

Shunt reduction by dedicated reduction device


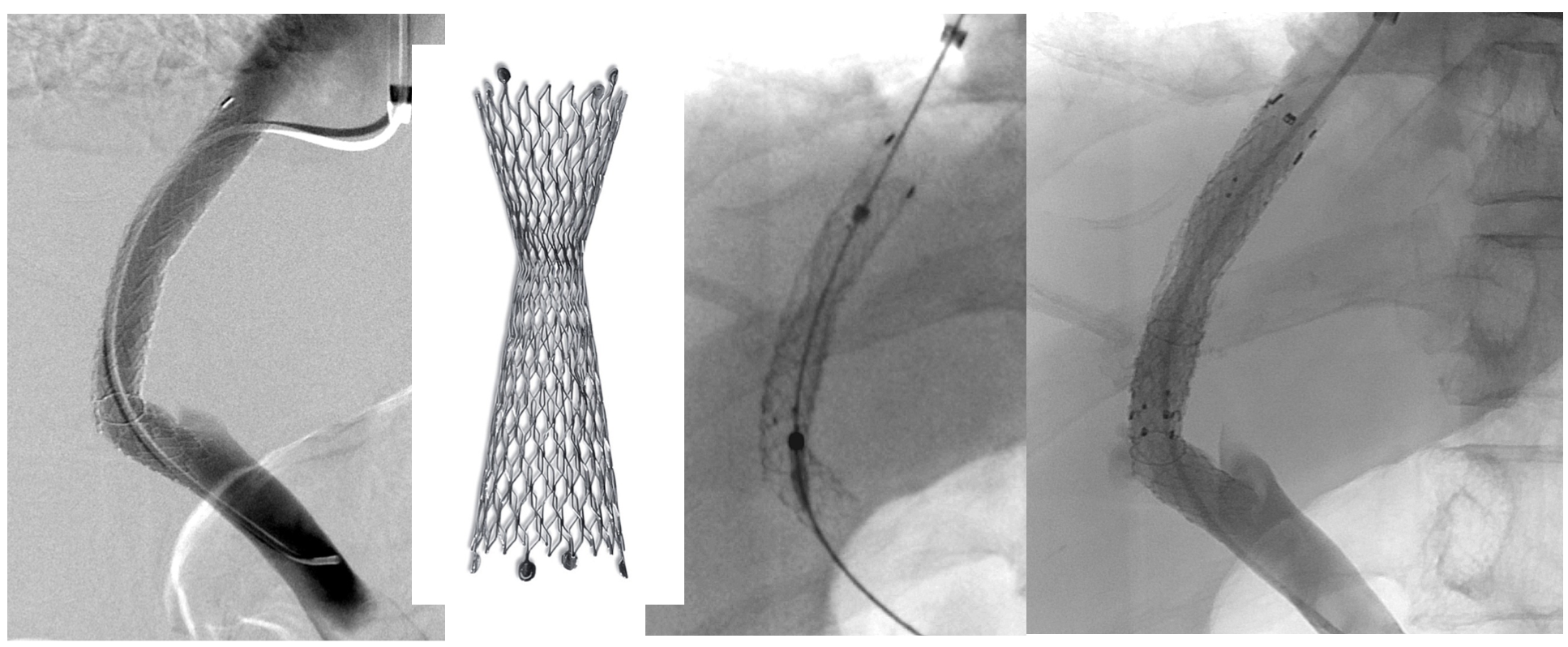


**Supp Figure 4.** Shunt reduction by dedicated reduction device (Sinus Reduction Stent®, Optimed, Ettlingen, Germany)


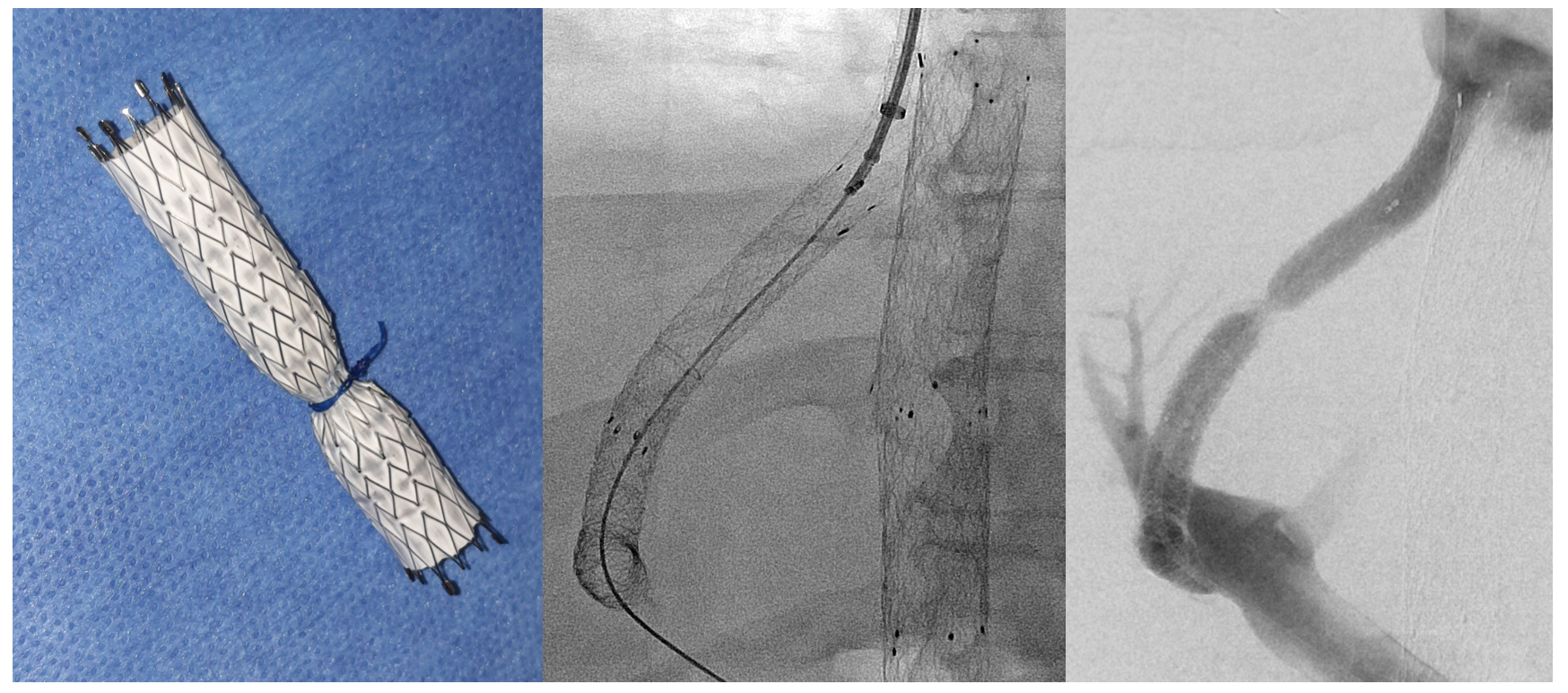


Shunt reduction with custom made reduction device

**Suppl Figure 5.** Custom made stentgraft: Fluency + Prolene suture. For preparation of the custom made devices, in a first step, a comercially available 10mm stentgraft (Fluency®, Bard, Murray Hill, NJ, USA) was half released from the introducer device and a 3.0 Prolene suture was twined around in order to create a hourglass shape with a predefined stenosis. In a second step, the prepared device was completely reloaded into a 10F sheath to cover the whole decive in order to serve as an outer introducer sheath of the whole thing and for final deployment. The preloaded 10 F sheath was advanced into the TIPS tract and the custom made device was deployed by pullback of both, first release of the preparated part of the stentgraft from the outer 10F sheath, and second a final release of the non-prepared half of the stentgaft from its original introducer device.


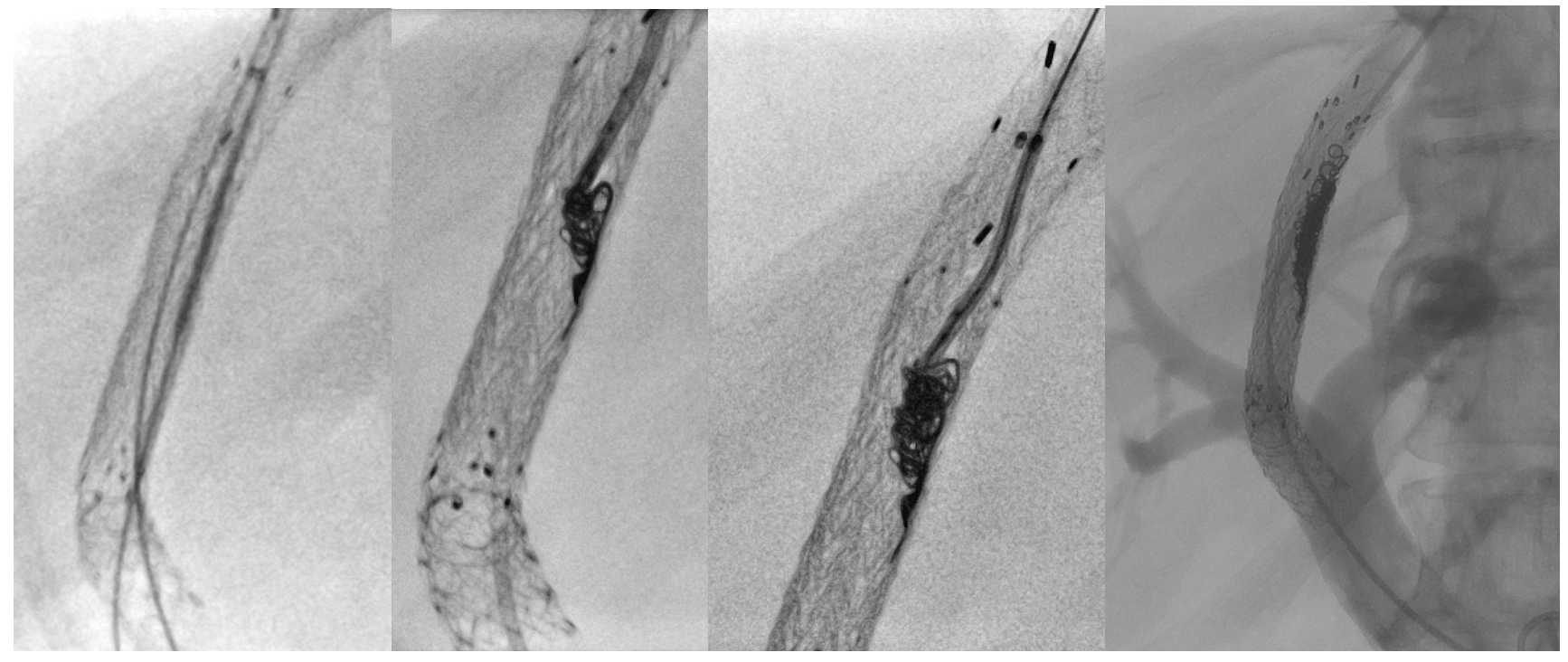


**Suppl Figure 6.** Fluency + Coiling of the interspace between the Viatorr stentgraft and the second stentgraft

Stentgraft in Viatorr and coiling of the interspace
